# Hepatitis C Virus Co-Infection Increases the Risk of Anti-Tuberculosis Drug-Induced Hepatotoxicity among Patients with Pulmonary Tuberculosis

**DOI:** 10.1371/journal.pone.0083892

**Published:** 2013-12-19

**Authors:** Nino Lomtadze, Lali Kupreishvili, Archil Salakaia, Sergo Vashakidze, Lali Sharvadze, Russell R. Kempker, Matthew J. Magee, Carlos del Rio, Henry M. Blumberg

**Affiliations:** 1 National Center for Tuberculosis and Lung Diseases, Tbilisi, Georgia; 2 Infectious Diseases, AIDS and Clinical Immunology Research Center, Tbilisi, Georgia; 3 Division of Infectious Diseases, Department of Medicine, Emory University School of Medicine, Atlanta, Georgia, United States of America; 4 Hubert Department of Global Health, Rollins School of Public Health of Emory University, Atlanta, Georgia, United States of America; 5 Department of Epidemiology, Rollins School of Public Health of Emory University, Atlanta, Georgia, United States of America; Temple University School of Medicine, United States of America

## Abstract

**Background:**

The country of Georgia has a high prevalence of tuberculosis (TB) and hepatitis C virus (HCV) infection.

**Purpose:**

To determine whether HCV co-infection increases the risk of incident drug-induced hepatitis among patients on first-line anti-TB drug therapy.

**Methods:**

Prospective cohort study; HCV serology was obtained on all study subjects at the time of TB diagnosis; hepatic enzyme tests (serum alanine aminotransferase [ALT] activity) were obtained at baseline and monthly during treatment.

**Results:**

Among 326 study patients with culture-confirmed TB, 68 (21%) were HCV co-infected, 14 (4.3%) had chronic hepatitis B virus (HBV) infection (hepatitis B virus surface antigen positive [HBsAg+]), and 6 (1.8%) were HIV co-infected. Overall, 19% of TB patients developed mild to moderate incident hepatotoxicity. In multi-variable analysis, HCV co-infection (adjusted Hazards Ratio [aHR]=3.2, 95% CI=1.6-6.5) was found to be an independent risk factor for incident anti-TB drug-induced hepatotoxicity. Survival analysis showed that HCV co-infected patients developed hepatitis more quickly compared to HCV seronegative patients with TB.

**Conclusion:**

A high prevalence of HCV co-infection was found among patients with TB in Georgia. Drug-induced hepatotoxicity was significantly associated with HCV co-infection but severe drug-induced hepatotoxicity (WHO grade III or IV) was rare.

## Introduction

The World Health Organization (WHO) estimates there were 8.6 million new cases of tuberculosis (TB) globally in 2012 and 1.3 million deaths due to TB[1]. TB is widespread in Georgia and other countries of the former Soviet Union and it has emerged as a major public health problem, including a high prevalence of multi-drug resistant TB (MDR-TB) [2-5]. In 2012, the incidence of TB in Georgia was 116 cases per 100,000 population [1] and the total TB case notification rate (new and re-treatment cases) was 158 cases per 100,000 population. High rates of MDR-TB have been reported from Georgia, one of 27 high MDR-TB burden countries as designated by the WHO [6]. A 2006 population-based survey carried out by our group found 7% of all new TB cases and 27% of retreatment cases were either MDR- or XDR-TB [4]. Recent data reported from the Georgian National TB Program indicated ~9% of new TB cases and ~31% of retreatment cases in 2012 had MDR-TB[1].

Hepatitis C virus (HCV) has also emerged as an important global public health problem. WHO estimates that 3% of the world’s population is infected with HCV and that more than 170 million chronic carriers are at risk of developing liver cirrhosis and/or liver cancer [7,8]. A high prevalence of HCV infection (6.9-7.8%) has been reported among blood donors in Georgia [9-11]. High-income countries have greatly reduced the incidence of transfusion-associated hepatitis; however, the risk of hepatitis from transfusion remains significant in low- and middle-income countries that have not fully implemented blood-screening measures. Incident cases of HCV also occur as a result of injection drug use (IDU) and through other means of percutaneous or mucous-membrane exposure including in health care settings where infection control measures are not fully implemented [8].

Globally, the prevalence of HCV infection among patients with TB has not been extensively investigated, and very limited data on rates of HCV co-infection among patients with TB exists. In Georgia, a previous study reported a high prevalence (22%) of HCV infection among patients with TB; in that study HCV co-infection was independently associated with previous incarceration, tattoo, and previous sexually transmitted infections [10].

Hepatotoxicity is the major adverse effect of three of the first-line anti-TB agents: isoniazid (INH), rifampin (RIF), and pyrazinamide (PZA). Underlying liver disease may increase the risk of developing drug-induced hepatotoxicity and there is concern that HCV and/or HIV co-infection may increase the risk of anti-TB drug-induced hepatotoxicity[12]. There remains very limited data on the impact of chronic viral hepatitis on the risk of incident anti-TB drug induced hepatotoxicity [12,13] and there are no previous data on the effect of HCV on anti-TB treatment, including hepatotoxicity, in Georgia. The purpose of our study was to assess risk factors for drug-induced hepatotoxicity among patients undergoing first-line anti-TB treatment and to determine in particular whether HCV co-infection increases the risk of anti-TB drug induced hepatotoxicity. A secondary objective was to determine the prevalence of HIV, HBV, and HCV co-infection (including distribution of HCV genotypes) among patients with TB in Georgia. 

## Methods

### Ethics Statement

The Institutional Review Board (IRB) at Emory University (Atlanta, GA, USA) and at the National Center for Tuberculosis and Lung Diseases (NCTBLD) (Tbilisi, Georgia) reviewed and approved the study. 

### Study Design, Population, and Setting

Consecutive patients with laboratory-confirmed, pulmonary TB were enrolled into this prospective cohort study at the Georgian National Center for Tuberculosis and Lung Disease after providing written informed consent. Eligible participants included newly diagnosed adult patients (>18 years of age) with pulmonary TB who had received <2 weeks of WHO-recommended directly observed short course (DOTS) therapy. Treatment included an intensive phase of four drugs (isoniazid [INH], rifampicin [RIF], pyrazinamide [PZA], and ethambutol [EMB]) for two months, followed by a continuation phase of RIF and INH for four months [14]. Fixed dose combinations of first line anti-TB drugs were given orally using WHO dosing recommendations based on patient weight bands [14,15]. Study subjects were seen at baseline and monthly during 6 months of treatment. At the baseline visit, patients were interviewed by a study investigator using a structured questionnaire that collected information on demographic, social, behavioral, and other patient characteristics. At the baseline visit 15 ml of blood was drawn to test for HIV, HBV (HbsAg and HbcAb), HCV, and liver enzymes (alanine aminotransferase [ALT], aspartate aminotransferase [AST], alkaline phosphatase [ALP], bilirubin and albumin [ALB]). At each monthly follow-up visit subjects provided 5 ml of blood for ALT monitoring. Those with baseline HCV co-infection received HCV genotype and viral load testing at baseline and after 2 and 6 months of therapy. Patients with symptoms suggestive of hepatitis or with an elevated ALT more than twice the upper limit of normal were referred to their physician for follow-up evaluation. 

### Laboratory Methods

AFB smear microscopy was performed using the Ziehl-Neelson staining method. Culture was performed using solid (Lowenstein-Jensen) and liquid (MGIT BACTEC 960) media using standard methodologies [16,17]. Drug susceptibility testing (DST) was performed by the Absolute Concentration method [18]. For serological testing blood samples were assayed for antibody to HIV-1 with the Abbott Recombinant HIV-1 assay (Abbott Laboratories, Abbott Park, IL, USA); samples reactive for HIV-1 were confirmed using a licensed western blot assay (DuPont Co, Wilmington, DE, USA). Antibody to HCV was assessed using the Ortho HCV Version 3.0 ELISA (Ortho Diagnostics Systems, Raritan, NJ, USA); the presence of Hepatitis B surface antigen (HBsAg) and core antibody (anti-HBcore) were assessed using the Auszyme Monoclonal and Corzyme assays (Abbott Laboratories, Abbott Park, IL, USA). Both HCV genotyping and HCV RNA levels (viral load) were performed by the Georgian Infectious Diseases, AIDS and Clinical Immunology Center, Tbilisi, Georgia. HCV RNA levels were performed on specimens from patients with a positive HCV antibody test. Genotyping was performed using the HCV Genotype Assay designed to identify HCV genotypes 1 to 6 (VERSANT® HCV Genotype 2.0 Products [LiPA] SIEMENS) [19]. 

### Definitions

Patients with a positive HCV antibody test were considered to be HCV infected (i.e., either past or current HCV infection). Hepatotoxicity was defined based on World Health Organization (WHO) guidelines—grade 1 hepatotoxicity: ALT level 51 to 125 U/L (1.25 to 2.5 times normal); grade 2: ALT level 126 to 250 U/L (2.6 to 5.0 times normal); grade 3: ALT level 251 to 500 U/L (5.1 to 10.0 times normal); grade 4: ALT level >500 U/L (>10 times normal), or ALT >250 U/L if accompanied by symptoms (e.g., nausea, vomiting, abdominal pain, jaundice) [20]. Incident hepatotoxicity was defined as an increase in 1 or more grades from baseline ALT level (during any of the 6 monthly follow up visits). Patients with hepatotoxicity at baseline (any grade) were considered incident hepatotoxic cases if their grade increased ≥1 levels during TB treatment. Time to incident hepatotoxicity was measured as the number of days from anti-TB therapy initiation to first increase in ALT grading. 

### Data Entry and Statistical Analysis

All study data were entered into an Epi-Info version 3.3.2 (Centers for Disease Control and Prevention [CDC], Atlanta, GA, USA) database. All statistical analyses were performed using SAS software version 9.3 (SAS institute Inc., Cary, NC, USA). For all analyses, a p-value <0.05 was considered statistically significant. Bi-variable associations between categorical variables and study outcomes were evaluated using the χ^2^ test or Fisher’s exact text; the Student’s t-test was used to compare differences in normally distributed continuous variables (means), and the Kruskal-Wallis test was used for comparison of non-normally distributed variables (medians). Multivariable logistic regression analyses were used to determine the independent risk factors for baseline HCV co-infection and to model the odds of incident hepatotoxicity. We considered previous literature, bivariate analyses, biologic plausibility, and directed acyclic graphs to determine potential confounders. Suspected confounders were included in multivariable models in order to estimate the adjusted effect on the outcome of each co-variable in the regression models. Product limit survival estimates were created using Kaplan-Meier survival curves and the log-rank test was used to evaluate statistical differences in the time to incident hepatotoxicity by HCV status. The analyses of incident hepatotoxicity were performed using Cox models to estimate hazard ratios and adjusted hazard ratios. Prior to performing hazard analyses, proportional hazards assumptions were tested for each risk factor. We used log-negative-log curves, goodness-of-fit tests (Schoenfeld residuals), and time-dependent models to assess proportionality of hazards over time.

## Results

Between March 2007 to March 2010, 346 newly diagnosed patients with TB were enrolled in the study. HCV serologic results were available for 326 (94.2%) patients; 20 patients without HCV serologic status were excluded from the final analyses. Study patients were all Caucasian (100%), predominantly ethnic Georgian (93%) and male (71%) and the median age was 37 years (range 21-92). At baseline, mean (standard deviation) ALT, AST, alkaline phosphatase (ALP), bilirubin, and albumin (ALB) were 48.9 U/L (171.1), 42.8 U/L (51.1), 94.5 (55.9), 0.59 (0.57), and 41.4 (9.1), respectively (Table 1). Also at baseline, 265 (81.3%) had normal ALT, and 61 (18.7%) had elevated ALT (grade 1 hepatotoxicity or greater). The median months on TB treatment was 6 (range 1-8) and 88% of patients enrolled into the study received ≥4 months of first-line anti-TB treatment.

**Table 1:** Baseline liver function measures and prevalent hepatitis C Virus (HCV) co-infection at time of new pulmonary tuberculosis (TB) diagnosis, Georgia 2007-2010, N=326.

	HCV (+) N=68	HCV (-) N=258	Total N=326
ALT* [units/L]			
Mean (STD)	47.7 (40.4)	49.2 (190.4)	48.9 (171.1)
Median (IQR)	37.0 (38.0)	24.0 (17.0)	26.0 (21.0)
AST* [units/L]			
Mean (STD)	57.0 (45.5)	39.2 (51.8)	42.8 (51.1)
Median (IQR)	43.0 (39.0)	28.0 (18.0)	30.0 (22.0)
ALP* [units/L]			
Mean (STD)	104.9 (57.2)	91.8 (56.6)	94.5 (55.9)
Median (IQR)	93.0 (51.0)	78.0 (40.0)	79.0 (44.0)
Bilirubin [mg/dL]			
Mean (STD)	0.59 (0.40)	0.59 (0.60)	0.59 (0.57)
Median (IQR)	0.50 (0.45)	0.50 (0.30)	0.50 (0.30)
ALB [g/dL]			
Mean (STD)	40.3 (10.2)	41.7 (8.8)	41.4 (9.1)
Median (IQR)	39.5 (13.0)	40.0 (13.0)	42.0 (13.0)
WHO Hepatotoxicity grade**			
N (%)	49 (72.1)	216 (83.7)	265 (81.3)
Normal	16 (23.5)	33 (12.8)	49 (15.0)
Grade 1	3 (4.4)	6 (2.3)	9 (2.8)
Grade 2	0	1 (0.4)	1 (0.3)
Grade 3	0	2 (0.8)	2 (0.6)
Grade 4	0	0	0

^*^ T-test or Kruskal-Wallis p-value ≤0.01

HCV=hepatitis C virus; ALT (U/L) = alanine aminotransferase, AST (U/L) = aspartate aminotransferase; ALP = alkaline phosphatase; ALB = albumin.

^**^ WHO Hepatotoxicity Grading levels as previously reported [20].

### Co-infection (HCV, HBV, and HIV)

At baseline, among the 326 study patients with TB, 68 (21%) were HCV co-infected, 14 (4.3%) had chronic hepatitis B virus infection (HBsAg+), and 6 (1.8%) were HIV co-infected. Among those with TB-HCV co-infection, 59 (86.8%) had viral load and genotype test results available; 12 patients had undetectable HCV viral load (and therefore could not have a genotype performed) and 47 patients had a detectable viral load. Genotype distribution was as follows: 21 (45%) type 1b, 11 (23%) type 2a/2c, 2 (4%) type 2a and 13 (28%) type 3a. In bivariable analyses, older age, male gender, alcohol use, current tobacco use, injection drug use (IDU), hepatitis B virus (HBV) co-infection, HIV co-infection, having a tattoo, history of incarceration (including living with an incarcerated person), and history of a sexually transmitted infection (STI) were significantly associated with baseline HCV co-infection (Table 2). In multivariable analysis, IDU (adjusted Odds Ratio [aOR]=14.2, 95% CI=5.2-38.8), current tobacco use/smoker (aOR=8.3, 95% CI=1.3-51.2), HBV co-infection (aOR=3.9, 95% CI=1.7-8.7), and having a tattoo (aOR=2.8, 95%CI=1.1-6.8) were independent risk factors for HCV co-infection at baseline among patients with TB (Table 3). 

**Table 2:** Factors associated with prevalent Hepatitis C Virus (HCV) co-infection at time of diagnosis of patients with pulmonary tuberculosis (TB), Georgia 2007-2010, N=326.

**Factor**	**HCV (+) n/N1 (%) 68/326 (20/9)**	**OR (95% CI)**	**p-value**
**Age**			
≥40	34/127 (26.8)		
<40	32/194 (16.4)	1.8 (1.1-3.2)	0.02
Missing	2/5 (20.0)		
**Gender**			
Male	65/230 (28.3)	12.2 (3.7-39.9)	<0.01
Female	3/96 (3.1)		
**Habitation**			
Urban	61/275 (22.2)		
Rural	6/42 (14.3)	1.7 (0.7-4.2)	0.31
Missing	1/9 (11.1)		
**Internally Displaced Person (IDP)**			
Yes	6/20 (30.0)		
No	60/286 (20.9)	1.6 (0.6-4.4)	0.40
Missing	2/20 (10.0)		
**Low education**			
Yes	51/214 (23.8)		
No	16/102 (15.7)	1.7 (0.9-3.1)	0.09
Missing	1/10 (10.0)		
**Alcohol Intake**			
Yes	48/166 (28.9)	2.8 (1.6-5.1)	<0.01
No	20/160 (12.5)		
**IDU**			
Yes	37/47 (78.7)	29.1 (13.1-64.4)	<0.01
No	30/266 (11.3)		
**Smoking**			
Yes	63/219 (28.8)		
No	4/97 (4.1)	9.4 (3.3-26.6)	<0.01
Missing	1/10 (10.0)		
**Evidence of HBV Infection (HBsAg+ and/or HBcAb+)**			
Yes	39/110 (35.5)	3.5 (2.0-6.2)	<0.01
No	29/216 (13.4)		
**HBsAg(+)**			
Yes	2/14 (14.3)	0.6 (0.1-2.8)	0.74
No	66/312 (21.2)		
**HIV+**			
Yes	4/6 (66.7)	8.0 (1.43-44.6)	0.02
No	64/320 (20.0)		
**Tattoo**			
Yes	31/58 (53.4)		
No	33/252 (13.1)	7.6 (4.0-14.3)	<0.01
Missing	4/16 (25.0)		
**History of Incarceration**			
Yes	22/29 (75.9)		
No	44/285 (15.4)	17.2 (6.9-42.7)	<0.01
Missing	2/12 (16.7)		
**Live with Prisoner**			
Yes	26/69 (37.7)		
No	37/241 (15.4)	3.33 (1.8-6.07)	<0.01
Missing	5/16 (31.3)		
**STIs**			
Yes	8/15 (53.3)		
No	58/295 (19.7)	4.6 (1.6-13.4)	<0.01
Missing	2/16 (12.5)		
**Blood Transfusion**			
Yes	6/21 (28.6)		
No	60/292 (20.5)	1.5 (0.5-4.1)	0.41
Missing	2/13 (15.4)		
**Surgery**			
Yes	15/66 (22.7)		
No	52/249 (20.9)	1.1 (0.5-2.1)	0.70
Missing	1/11 (9.1)		

**Table 3:** Multivariable logistic regression analysis for prevalent hepatitis C virus (HCV) co-infection at time of pulmonary tuberculosis (TB) diagnosis, Georgia, 2007-2010.

**Factor**	**aOR**	**95% CI**	**p-value**
IDU	14.2	5.2 - 38.8	<0.01
History of Cigarette Smoking	8.3	1.3 - 51.2	0.02
Evidence of HBV Infection (HBsAg+ and/or HBcAb+)	3.9	1.7 - 8.7	<0.01
Tattoo	2.8	1.1 - 6.8	0.02
HIV(+)	6.1	0.6 - 60.9	0.12
History of Incarceration	1.8	0.5 - 6.6	0.35
STI	1.5	0.3 - 7.4	0.62
Male	1.5	0.4 - 6.6	0.55
Low education	1	0.4 - 2.5	0.99
Age>40	0.9	0.4 - 2.2	0.95
Alcohol Intake	0.9	0.4 - 2.5	0.97

aOR = adjusted Odds Ratio; CI=Confidence Interval; IDU = Injection Drug Use; Evidence of HBV Infection = indicates chronic HBV infection (HBsAg+) and/or evidence of prior HBV infection (HBcAb+); HIV+ = Human Immunodeficiency Virus co-infection; STI=History of sexually transmitted infection(s); Low Education=Less than 9 years of school; Alcohol Intake = >3 drinks of alcohol intake daily. The logistic model contained all variables listed in the table.

### Incident Hepatotoxicity

**Figure 1:**
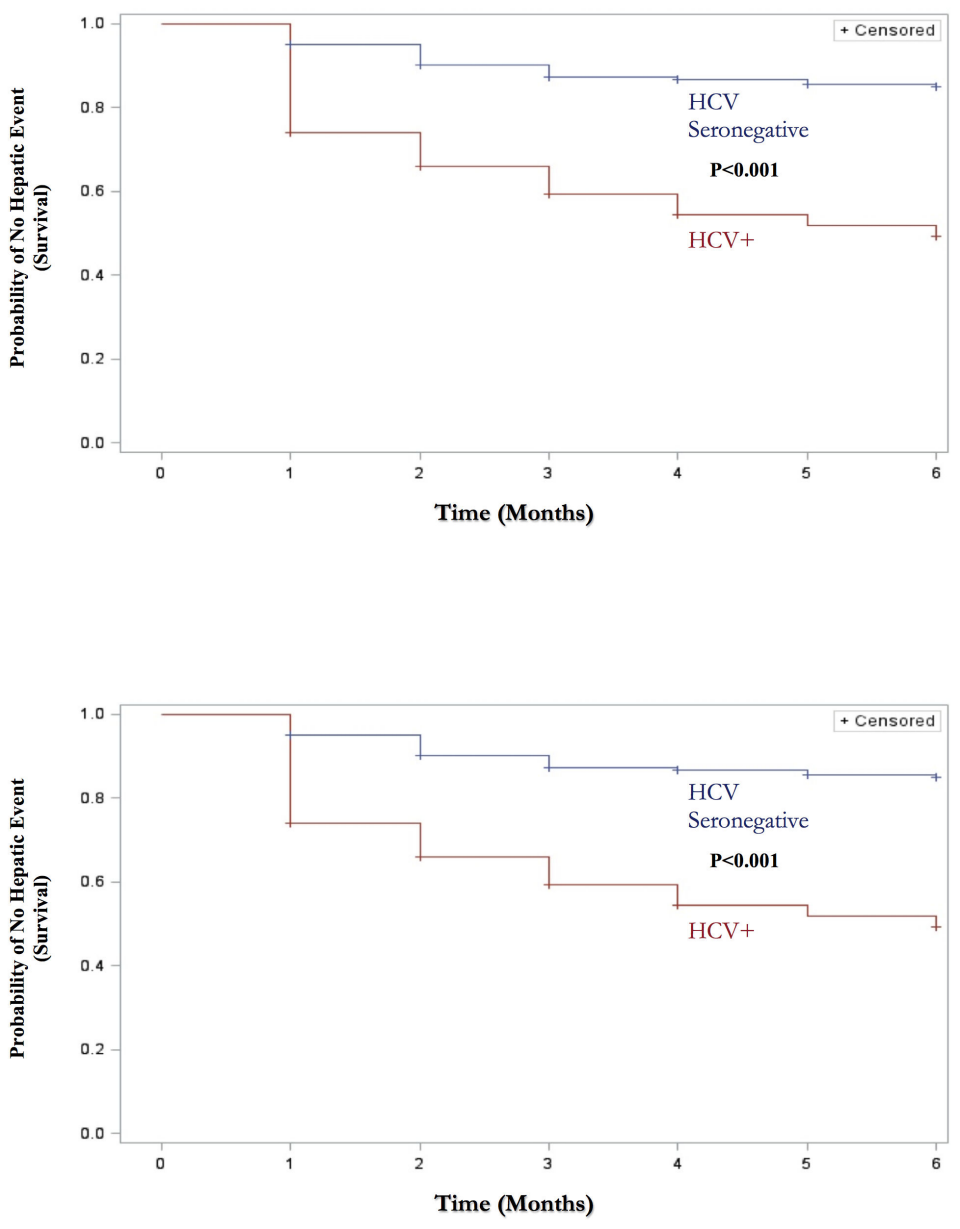
Time to incident hepatotoxic events among newly diagnosed patients with tuberculosis (TB) stratified by Hepatitis C Virus (HCV) status during 6 months of first-line anti-TB drug treatment, Georgia 2007-2010, N=326.

Study patients that did not return for any follow up visits (n=38) were excluded from incident hepatotoxicity analyses. Overall, 54 (18.8%) of the remaining 288 TB patients developed incident hepatotoxicity; 42 patients developed grade 1 toxicity (14.6%), 8 developed grade 2 (2.8%), 4 developed grade 3 (1.4%), and no patients developed grade 4 hepatotoxicity. Comparing patients with TB that had incident hepatotoxicity to those without, bivariable analyses found that HCV co-infection (43.9% vs. 12.6%), IDU (39% vs. 14.5%), history of incarceration (34.6% vs. 16.8%), being a current smoker (23.2% vs. 13.6%) and male gender (22.6% vs. 9.5%) were significantly associated with incident hepatitis during the first six months of first-line anti-TB treatment (Table 4); there was a trend for HBsAg positivity (41.7% vs. 17.8%). In multivariable analysis, HCV co-infection (adjusted Hazards Ratio [aHR]=3.2, 95% CI=1.6-6.5) was significantly associated with incident toxicity among patients with active TB disease and there was a trend for chronic HBV infection (HBsAg+) (aHR=2.4, 95% CI=0.9-6.4) (Table 5). Compared to TB patients not infected with HCV, Kaplan-Meier survival curves showed that time until developing hepatotoxicity was shorter among TB patients with HCV co-infection (p<0.01) (Figure 1). None of the patients interrupted or terminated the treatment due to the hepatotoxicity symptoms or signs. Results of subgroup bivariable analyses (HCV co-infected patients only) for incident hepatotoxicity by detectable viral load and genotype are shown in Table 6. Among patients with TB and HCV co-infection, no difference in the proportion of patients with incident hepatotoxicity was found by detectable viral load (p=0.62) or genotype (p=0.93).

## Discussion

A high prevalence of HCV (21%) co-infection and lower prevalence of chronic HBV infection (4.3%) and HIV (1.8%) co-infection were found among newly diagnosed patients with pulmonary TB in Georgia. The most prevalent HCV genotype among newly diagnosed patients with TB was type 1b (45%); HCV genotypes 2a/2c, 2a, and 3a were less common among patients with TB. We also found that prevalent HCV co-infection was independently associated with IDU, current smoking, and HBV co-infection. Incident hepatotoxicity was common during the 6 months of follow-up in this study with approximately one-fifth of TB patients developing hepatotoxicity (grade 1 toxicity or greater) during anti-TB therapy. Importantly, we found in multivariable analysis that HCV infection was independently and significantly associated with incident hepatotoxicity. There was a trend for chronic HBV infection (HBsAg+) but the analysis for HBV was limited by the relatively small number of study subjects with chronic HBV infection.

Few studies have examined the impact of HCV infection on incident hepatotoxicity during anti-TB treatment [12,21-23]. There has been concern that underlying chronic liver disease caused by viral hepatitis increases the risk of first-line anti-TB drug-induced hepatotoxicity. In our study, 18.8% of subjects with a normal baseline ALT level developed hepatotoxicity during the treatment, indicating an important increase from baseline ALT level during the six months of anti-TB therapy. Among patients with HCV co-infection, 43.8% developed hepatotoxicity, nearly double the proportion of chronic hepatitis patients with incident hepatotoxicity recently reported by Park et al in Korea [13]. Patients with active TB disease and HCV co-infection in our study had a similar HCV genotype distribution to that previously reported in Georgia [19]; however, we found no differences in incident hepatotoxicity by HCV genotype. 

**Table 4:** Bivariate analysis of risk factors for incident hepatotoxicity during 6 months of anti-tuberculosis (TB) treatment among newly diagnosed patients with pulmonary tuberculosis in Georgia, 2007-2010, N=288.

**Risk Factor**	**Incident hepatotoxicity n/N (%) 54/288 (18.8)** ^1^	**Hazard Ratio (95% CI)**	**p-value**
HCV +			**<0.01**
Yes	25/57 (43.9)	4.0 (2.3, 6.8)	
Grade 1	19/57 (33.3)		
Grade 2	5/57 (8.8)		
Grade 3	1/57 (1.8)		
Grade 4	0/57 (0)		
No	29/231 (12.6)	1.0	
Grade 1	23/231 (10.0)		
Grade 2	3/231(1.3)		
Grade 3	3/231 (1.3)		
Grade 4	0/231 (0)		
HBsAg+			
Yes	5/12 (41.7)	2.4 (0.9, 5.9)	0.07
No	49/276 (17.8)	1.0	
Age (years)			
≥40	16/101 (15.8)	0.9 (0.5, 1.6)	0.64
<40	37/182 (20.3)	1.0	
Missing**^*2*^**	1/5 (20.0)		
Gender			
Male	46/204 (22.6)	2.7 (1.3, 5.6)	**0.01**
Female	8/84 (9.5)	1.0	
Place of residence			
Urban	44/245 (18.0)	0.7 (0.3, 1.6)	0.45
Rural	7/34 (20.6)	1.0	
Missing**^*2*^**	3/9 (33.3)		
Internally displaced person			
Yes	2/18 (11.1)	0.5 (0.1, 2.2)	0.37
No	48/250 (19.2)	1.0	
Missing	4/20 (20.0)		
Alcohol intake			
Yes	29/143 (20.3)	1.3 (0.7, 2.2)	0.39
No	25/145 (17.2)	1.0	
IDU			
Yes	16/41 (39.0)	2.9 (1.6, 5.1)	**<0.01**
No	34/234 (14.5)	1.0	
Missing	4/13 (30.8)		
Current Smoker			
Yes	32/138 (23.2)	1.8 (1.0, 3.1)	**0.03**
No	19/140 (13.6)	1.0	
Missing	3/10 (30.0)		
HIV+			
Yes	1/4 (25.0)	1.4 (0.2, 9.8)	0.77
No	53/284 (18.7)		
Tattoo			
Yes	12/46 (26.1)	1.5 (0.8, 2.9)	0.19
No	38/226 (16.8)	1.0	
Missing**^*2*^**	4/16 (25.0)		
History of Incarceration			
Yes	9/26 (34.6)	2.2 (1.1, 4.4)	**0.04**
No	42/250 (16.8)	1.0	
Missing**^*2*^**	3/12 (25.0)		
MDR-TB			
Yes	10/37 (27.0)	1.5 (0.8, 3.0)	0.23
No	42/240 (17.5)	1.0	
Missing**^*2*^**	2/11 (18.2)		
XDR-TB			
Yes	1/6 (16.7)	0.7 (0.1, 5.3)	0.74
No	51/271 (18.8)	1.0	
Missing**^*2*^**	2/11 (18.2)		
Sexually transmitted infection(s)			
Yes	3/13 (23.1)	1.4 (0.4, 4.4)	0.60
No	46/260 (17.7)	1.0	
Missing**^*2*^**	5/15 (33.3)		

Abbreviations: CI=Confidence Interval; HCV(+)=Hepatitis C Virus co-infection; HBsAg(+)=hepatitis B surface antigen positive (chronic Hepatitis B Virus infection); HIV(+) = Human Immunodeficiency Virus co-infection; Alcohol Intake = >3 drinks of alcohol intake daily; IDU = Injection Drug Use; MDR-TB = Multidrug resistant Tuberculosis; XDR = Extensively drug resistant tuberculosis.

^1^ (n/N) n-patients with incident hepatotoxicity/N-all new TB patients at risk of hepatotoxicity; patients with baseline hepatotoxicity were excluded from incident analyses (n=61).

Note: Patients with missing data were coded into the null category (1.0) for each of the hazard ratio estimates.

**Table 5:** Multivariable analysis of risk factors for incident hepatotoxicity among newly diagnosed patients with pulmonary tuberculosis in Georgia, 2007-2010, N=288.

**Risk Factor**	**aHR^*1*^**	**95% CI**	**aHR p-value**
HCV+	3.2	1.6, 6.5	<0.01
HBsAg+	2.4	0.9, 6.4	0.07
Male	1.9	0.8, 4.9	0.15
Age ≥40 years	0.7	0.4, 1.3	0.21
IDU	1.4	0.6, 3.0	0.47
History of incarceration	0.8	0.3, 2.0	0.63
Alcohol intake	0.8	0.4, 1.5	0.50
Current smoker	1.2	0.6, 2.3	0.60
HIV+	0.7	0.1, 5.6	0.72

Abbreviations: aHR=adjusted hazard ratio; CI=Confidence Interval; HCV(+)=Hepatitis C Virus co-infection; HBsAg(+)=hepatitis B surface antigen positive (chronic Hepatitis B Virus infection); HIV(+) = Human Immunodeficiency Virus co-infection; Alcohol Intake = >3 drinks of alcohol intake daily; IDU = Injection Drug Use

^1^ Adjusted for all risk factors in the table. Patients with missing data for risk factors were coded into the null category (1.0) for each of the adjusted hazard ratio estimates.

**Table 6:** Incident hepatotoxicity among hepatitis C (HCV) co-infected patients with newly diagnosed pulmonary tuberculosis (TB), Georgia 2007-2010, N=53.

	**Incident Toxicity N=25 (47.2%) N (%)**	**No Toxicity N=28 (52.8%) N (%)**	**Total N=53 N (%)**	p-value*
**HCV Viral Load**				
Detectable	21 (49.0)	22 (51.0)	43 (81.1)	0.62
Undetectable	4 (40.0)	6 (60.0)	10 (18.9)	
**HCV Genotype**				
1b	9 (36.0)	11 (39.3)	20 (37.7)	
2a	1 (4.0)	1 (3.6)	2 (3.8)	
2a/2c	5 (20.0)	5 (17.9)	10 (18.9)	0.93
3a	6 (24.0)	5 (17.9)	11 (20.8)	
Undetectable	4 (16.0)	6 (21.4)	10 (18.9)	

^*^ Chi-square p-value for general association

Injection drug use is a primary risk factor for the transmission of HCV globally and in Georgia [24,25]. Our finding that having a tattoo was a risk factor for HCV is also consistent with previous studies conducted among patients with TB [10]. In Georgia, we suspect that the majority of commercial tattoo facilities share poor infection control practices and hygienic prison tattoo programs are not available. Tattoo artists in Georgia do not require a license. Accredited education programs that emphasize the importance of proper hygiene and the risk of blood-borne transmission infections by tattoo may be beneficial for both the civilian and penitentiary sectors. A recent systematic review recommends proper trainings of tattoo artists and urges clinicians to consider screening for HCV among those who have recently received tattoos or have a history of receiving one [26].

While the development of hepatitis among patients on anti-TB drugs was common and an increased risk was seen among those with chronic hepatitis infection, severe hepatotoxicity (grade 3 or grade 4) was distinctly uncommon and did not impact clinical care. Discontinuation of first-line TB treatment due to hepatotoxicity was not reported in our study. Isoniazid (INH), rifampin, and pyrazinamide are all potentially hepatotoxic. Some previous studies have suggested that INH is likely the most important drug warranting discontinuation of therapy (hepatotoxic discontinuation proportion range from 0.1% to 10%) while other reports indicate that pyrazinamide causes more hepatotoxicity than INH or rifampin [27,28]. Current treatment guidelines from the American Thoracic Society/Centers for Disease Control (CDC)/Infectious Diseases Society of America recommend that if there if AST (or ALT) levels are more than five times the upper limit of normal (with or without symptoms) or more than three times the upper limit of normal in the presence of symptoms, hepatotoxic drugs (e.g., INH, rifampin, and pyrazinamide) should be discontinued and the patient evaluated carefully [29]. Only 4 participants in our study had >5 times the upper limits of normal ALT values. The vast majority of patients with hepatotoxicity in our study developed mild to moderate (Grades 1 and 2) hepatitis and all of the participants who developed incident hepatitis during the treatment were asymptomatic and did not require treatment interruption or termination. A prior report [12] suggests that patients with HCV and HIV co-infection are at increased risk of anti-tuberculosis drug induced hepatotoxicity compared to the risk posed by infection with a single virus. However, given the low prevalence of HIV infection in our patient population, we were not able to assess whether there is an increase risk from dual HCV and HIV infection. 

There were limitations to our study. We measured hepatotoxicity at monthly intervals during anti-TB treatment and some patients may have developed hepatotoxicity within the interval and later resolved to a normal level. Consequently our estimate of the proportion of patients who developed hepatotoxicity may be underestimated. Secondly, we did not have data to assess TB treatment adherence by individual patient and therefore could not assess the influence of treatment adherence on incident hepatotoxicity. However, all patients in the study were treated with directly observed therapy and the large majority of patients received the first two months of treatment as an inpatient (as is a standard practice in Georgia to give the intensive phase as an inpatient). Thirdly, liver function tests can fluctuate over time in patients with HCV infection and it may not be possible to differentiate between hepatotoxicity due to the anti-TB drugs and fluctuations in ALT over time due to underlying chronic active hepatitis.

## Conclusions

Our study documented a high prevalence of HCV (21%) co-infection and lower prevalence of chronic HBV (4.3%) or HIV (1.8%) co-infection among newly diagnosed patients with laboratory confirmed TB in the country of Georgia. Patients with HCV co-infection undergoing treatment with first line anti-TB drugs were more likely to develop drug-induced hepatotoxicity. Overall, we observed a low risk of severe (Grade 3 or 4) incident hepatotoxicity, even among TB patients with HCV co-infection. Therefore, among patients with active TB disease with HCV co-infection and normal baseline liver function test levels, routine monitoring of by monthly serum alanine aminotransferase [ALT] activity during first-line anti-tuberculosis therapy does not appear to be warranted based on data from our study. 
